# Prediction of the impact on air quality of the cities receiving cruise tourism: the case of the Port of Barcelona

**DOI:** 10.1016/j.heliyon.2019.e01280

**Published:** 2019-03-14

**Authors:** Ignacio Ruiz-Guerra, Valentín Molina-Moreno, Francisco J. Cortés-García, Pedro Núñez-Cacho

**Affiliations:** a University Complutense of Madrid, Spain; b University of Granada, Spain; c Universidad Autónoma de Chile, Chile; d University of Jaén, Spain

**Keywords:** Environmental science

## Abstract

The cruise tourism industry has experienced significant growth in recent years and has a very positive outlook for the future. However, its environmental impact requires a review of its sustainability, including the analysis of its social, economic and environmental balance, and the evaluation of its impact on port cities receiving cruise ships. The purpose of this document is twofold: First, to analyze the relationship between air quality, as an environmental variable, and the volume of cruise ships and passengers that visit a port with the aim of generating information. Secondly, it is intended to develop an index, based on the information already available that allows cities to predict the impact of this activity, so that decisions are made to alleviate these effects. Methods: The primary data taken monthly for the period 2006–2017, related to the level of emissions and the number of cruises and passengers, are used as a basis. A regression analysis is performed to determine the relationship between air pollution and the number of tourists coming from the cruise ships. As a contribution, the results show the influence of this type of tourism on environmental indicators. In addition, in an original way, a regression function is established that allows estimating the future impact of the cruise industry in the ports cities. So that, cities can prevent this type of environmental impact. This will make it easier for the cruise tourism industry to develop more sustainable models in the long term.

## 1 Introduction

Over the last few decades, the magnitude of the cruise tourism industry has experienced increased more greatly than the global tourism industry. From 1980 to 2016, the growth of this industry in the Mediterranean area was continuous, as stated by the Florida-Caribbean Cruise Association/FCCA [1]; Cruise Lines International Association (CLIA) [2], and Chang et al. [3]. Not only has the number of operations grown, but also the number of routes and the volume of passengers have increased, necessitating a series of changes to the tourism sector [4, 5, 6]. In the current year, the existing forecasts regarding the evolution of the number of passengers have already been exceeded. Specifically, in 2014, it was estimated that there would be over 25 million passengers in the years 2019–2020 [1,6,7]. However, in 2017, by June there had already been 25.3 million passengers. The growth of the cruise tourism industry worldwide expanded in the first decade of the 21st century, but also witnessed a change in positioning scenarios for the cruise product, that is, the volume of the cruise markets has been transferred between continents, with the United States moving from a market share of 70%–56%; and Europe increasing its share from 21% to have 30% by 2011, causing it to become the second most important world market. Other world destinations have increased their market share from 9% to 14% [2], and there have been more than 22 million passengers throughout the world. Predictions based on information from the Cruise Market Observatory suggest that the cruise tourism industry will reach 25 million in 2019, visiting nine million Spanish ports.

Since the 1970s, the cruise tourism industry has experienced a strong increase in activity, although with less development than other branches of the tourism industry, such as accommodation, or air transport [8, 9, 10]. Green development has been of particular interest to a range of industries worldwide, including the air transportation industry [11], bus transportation [12], and the cruise tourism industry [13]. Accordingly, a series of responsibilities towards the environment are required (Declaration of San José), which means that these industries have to focus on three key aspects: economic benefits, environmental commitment, and maintenance of the cultural integrity of the countries involved. These three complementary aspects help us to understand the notion of sustainable tourism.

The growth of the cruise tourism industry has come from the restructuring of old ships from North America and other parts of the world, including Europe [14]. In recent years, not only has machinery been reused, but also, the growth of the sector has generated large investments in new and larger ships. There has been a shift from those that can accommodate 1000 passengers, to the current ones that can house, generally, between 4.000 and 6.000 passengers [15]. Therefore, cruise tourism industry can be considered to be a mature sector that is affected by macroeconomic factors such as epidemics, the geopolitical situation of destinations, and economic crises. These factors cause changes in the strategic decisions made by shipping companies [16, 17, 18].

The cruise tourism industry is continuously devising innovative strategies to generate greater possibilities for business development and to improve and expand the activities on board. Innovation is not only based on the design of the boats, but also on the products that are offered, such as increasingly specialized itineraries, new ports of call, and new base ports. Shipping companies have been able to offer customized products in a market-scale context [19]. Life on the ships not only satisfies a diverse range of consumers, but also maximizes the passengers' expenses on board [20].

Several works have considered the economic viability of the cruise tourism industry. For example, Garay, Canoves, and Pratt [21] analyzed the economic impact of the cruise industry in the city of Barcelona. Their study of the socioeconomic impact of cruise ships around the urban zone ports suggested that cruise passengers spend more in these areas of the port, usually in the more important ports (base ports), rather than in the ports of call. Just as the shipping companies are making great efforts to capture, through innovation, greater expenses within the ship itself, generating complete products that maximize cruise passenger spending, preferably during navigation, should be developed, rather than in the ports of destination [22].

All the positive evolution of the cruise industry can be compromised as the cities receiving the cruises increase their environmental demands. The boats that do not verify certain conditions cannot be dedicated to the industry and this will have an impact on the future of the sector. Therefore, a key aspect for the future of the industry is the control of its environmental impact in the host cities. The rulers of the cities need to know what the environmental impact of this industry is. Several aspects have been analyzed, generally through case studies, which relate the impact of cruises with the cities visited. However, we must advance in the concrete knowledge of this impact, calculate its magnitude, know which environmental indicators are most affected and predict future behavior. Of the different impacts, we have focused on environmental pollution, which is one of the most worrisome issues due to CO2 emissions, PM10 and PM2.5 particles, NO2 and SO2.

Ground-level ozone (O3) and particulate matter (PM) are pollutants that adversely affect human health. In this way, exposure to these air pollutants generates great concern in urban areas, with high population density and very exposed to polluting factors [23, 24, 25, 26]. These particles generate respiratory problems and cardiovascular health. Short-term respiratory impacts range from reduced lung function, cough, and throat irritation to asthma attacks. The long-term impacts of air pollution include chronic obstructive pulmonary disease (COPD) and cardiovascular health disease [27]. Besides, according to EU Environment Agency, the emissions of the main air pollutants in Europe are: Sulphur oxides: SO2; nitrogen oxides: NOx; ammonia: NH3; non-methane volatile organic compounds: NMVOCs; fine particulate matter: PM2.5 [28].

To become a sustainable activity, the cruise tourism industry needs to balance both environmental impacts and benefits to transition towards a more sustainable tourism model. This includes the reduction of environmental impact. For this reason, our research goal was to analyze the relationships between cruise industry activity and indicators of the environmental impact of this tourist activity on host cities. In this way, we will analyze one of the main negative impacts of this industry, covering the gap that has been detected in the literature review. To reach our objective, we analyzed the case of the Spanish port of Barcelona, one of the busiest in the cruise industry in Europe. Specifically, our research questions were as follows:

**RQ**_**1**_: What is the relationship between the number of passengers visiting the port and the environmental impact on the air quality of the port city?

**RQ**_**2**_: Could be predicted the environmental impact of the cruise industry on the air quality of the receiving cities? What is the function that allows this prediction?

The information will also allow adopting the corresponding measures aimed at converting this type of tourism into a sustainable model according to the requirements of society, thus ensuring its viability in the long term.

## 2 Theory

The industry can be considered to be an ecosystem [29, 30, 31]. Thus, in the current paper we propose the application of human ecology theory to the study of the interactions and interdependence between the cruise tourism industry and the environment to create an ecological framework, the Industrial Ecology theory, for decision-making and managing company resources [31, 32, 33, 34, 35].

The Industrial Ecology theory is a synthesis of assumptions, concepts, and propositions of ecology in various disciplines and the general theory of the system that refers to the “creation, use, and management of resources for adaptation, human development, and sustainability of environments”. It can be used to describe and explain, not only the interactions that occur within the industries, but also their transactions with the environment. This double perspective is based on science, but applies the principles, methods, and results to daily activities [36]. For this reason, this theory is adequate for the analysis of both the cruise industry and its environmental impact. These considerations will allow the cruise industry to manage its relationships with the community to avoid negative perceptions.

Regarding to the principles and core values of this theory, it be assuming three principles as starting point: Firstly, the cruise Industry interacts with its environment by creating an ecosystem, in which, according to systems theory, the parts and the whole are interdependent. Physical, biological and social laws guide the interactions that occur within this ecosystem and the industry continually requires matter and energy from the ecosystem. Secondly, the cruise industry performs functions of physical, biological and economic maintenance, for its members, for itself, and for the common interest of the whole macro system. Thirdly, there is interdependence between cruise industry and resources. Thus, ecological wealth depends on the decisions and actions of companies. The well-being of this industry cannot be separated from the well-being of the whole ecosystem [19].

Therefore, the industry must find the balance between the demands of the ecosystem and those of individual industries as a whole. The core values of the theory are the survival of the business, the sustainability of environment, looking simultaneously the “betterment” of the individual situations.

Decision-making is the central process through which the cruise industry directs its actions to achieve their goals. Collectively, industry decisions and actions have an impact on society, culture and natural environment. The whole system consists of the cultural norms, and the beliefs that influence the other systems, being the framework for legislative changes. An ecological transition occurs when a person's position in the ecological environment is altered as a result of a change in paper, on stage or in both [17, 20]. So, there are relevant questions regarding to cruises industry that can be addressed by this theory, such as:

• What are the processes through which the cruises industry ensures survival, improvement of the quality of life, and the sustained performance of natural resources?
• How does the industry identify, access, and transform resources through creative activities of organization and sustenance?
• What is the relationship between cruises industry, social values, and the consumption of resources?
• How does the cruise industry decide the routes and how the resources used will be transferred to the environment?
• How do the sociocultural milieu and values influence the decisions made and the actions carried out by the cruise industry?
• What can be done to improve the quality of life and preserve the environment and resources needed for life?
• Could we predict the effects of the Cruises industry in the cities?

The idea of responsible tourism originates from the movement for the sustainable development of tourism. Sustainability was defined in 1987 by the Brundtland Commission as "development that meets the needs of the present without compromising the ability of future generations to meet their own needs" (World Commission on Environment and Development [WCED], 1987). Klein [37] states that sustainable tourism maintains the ESG (environmental, social governance) approach and companies participating in the industry are expected to do what is morally and ethically "correct" [38] from the perspective of clients and communities. Therefore, according to the Industrial ecology theory, responsible tourism seeks, among other aspects, to minimize negative economic, environmental and social impacts.

The challenges of implementing the principles of sustainability in practice relate to the broad and widely interpreted nature of the concept. In general, it is accepted that sustainability respects three basic aspects (protection of the environment, economic development, and social equity), including how to protect the viability of these principles for future generations [39]. According to Kuhlman and Farrington [40], sustainability can be defined as the maintenance of well-being for a long period, thinking about an indefinite period. This largely covers the environmental dimension of the triple bottom line, but environment and sustainability are not synonymous. On the one hand, some forms of environmental degradation are reversed relatively easily and are highly harmful in the present, for example, many forms of air and water pollution.

Since the publication of the Brundtland Report, sustainable tourism has sought to achieve developments that meet our needs, but do not compromise the environment for future generations (1987). Accordingly, a number of countries, via the UN, agreed on a Program of the World Commission on Development and Environment, with the World Tourism Organization (UNWTO). For tourism sector, 2017 was the year of Sustainable Tourism [7]. Likewise, the countries themselves have sought not only their own commitment but have also promoted actions to make companies more ethical and careful regarding the environment.

The reduction of the negative effects of tourism that these commitments imply assists with the understanding of the concept of responsible tourism promoted by Responsible Tourism in Destinations, 2002, which is based on these aspects:

• Minimizing its negative impact on economic, environmental, and social aspects;
• Increasing the economic benefits generated to the local communities, since it allows them to access new conditions for the implementation of employment opportunities;
• Involving the local community in making decisions about what affects their way of life; and
• Contributing positively to the conservation of natural and cultural heritage.

As sustainability becomes increasingly important in risk management and reputation, it is possible that several of the major cruise companies must reconsider their current approach to sustainability reporting if they wish to preserve, and ideally improve, their position within a very competitive market. Jones, Hillier and Comfort [41] note that current commitments to sustainability within the cruise industry and commitments are driven primarily by commercial imperatives, seeking to improve their efficiency in a broad range of economic, social and environmental issues rather than maintain the viability and integrity of natural ecosystems and reduce the demands of finite natural resources. They point out that the main business models of the cruise industry are focused on continuous growth and consumption, which implies a weak approach towards sustainability. Caric [5] is also critical with the situation of sector indicating that cruise tourism is far from the concept of sustainability or sustainable tourism as the world tourism organization promotes it [8] so it is of all concern and responsibility transform the current model to ensure the survival of the cruise industry.

On the other hand, environment protection as a path leading to sustainability is never ending process [42]. Carić and Mackelworth [43] note that the environmental impact of the cruise industry is largely due to marine diesel engines that use heavy fuels rich in sulfur and ash-forming metals [44]. The intensity of air pollution from fuel combustion depends on the activity of the ship. If the ship is in the open sea, maneuvering, or in the dock, the gases emitted will vary, but always consist of NOx, SOx, COx, O3 and suspended particles (PM). It is estimated that the overall contribution of ship emissions is 15% and 9% of NOx and SO2, respectively [45]. Within the Mediterranean, cruise and CO2 emissions from passenger ships are estimated at up to 10% of all ship emissions [46]. This makes the cruise the largest CO2 contributor to tourism [47].

Atmospheric emissions can have localized and regional effects on the environment. The Mediterranean has been identified as one of the largest shipping routes and the most polluted regions in the world, which is exacerbated in the hot summer months [48]. Considering the potential local effect of air pollution there must be concerns about the potential damaging effect moored cruise ships could have on the city and its general ability to attract all forms of tourism. All these environmental costs are very difficult to calculate since the cruise industry is, in general terms, an unregulated activity and it is difficult to measure its impact globally [49].

In this work, we focused on the development of the commitment to environmental impact reduction through the study of the negative environmental externalities related to the cruise industry, not only in terms of the destinations, but also for the planet. We started from the few previous studies [50], that have researched sectors such as accommodation and air transport, but not cruise companies [1, 32, 51, 52, 53, 54]. It is important to be able to calculate the environmental impact of cruise companies to identify cost saving opportunities and demonstrate, through social responsibility commitments, the commitment of these companies.

The analyses of emissions derived from the cruise industry have frequently measured the amount of waste generated. For years, cruise companies have assumed that the only exit from the industry was their dissolution [55, 56, 57, 58, 59, 60, 61, 62]. These analyses did not include other aspects, such as the unloading of wastewater generated by ships (with the approval of international organization directly into the sea, affecting the marine fauna and flora, nor did they take into consideration the impact on the environment of the port cities.

On the other hand, the emissions produced by the operation of cruise ships due to the increase in cruise traffic has led to an increase in pollution, not only at stopover destinations, but also during the journey [63]. According to the 2007 estimations by the US EPA, a category 3 ships that operates in the Exclusive Economic Zone of the United States, emits a total of 870,000 tons of nitrogen oxide. Consequently, the cruise industry is trying to reduce the emission levels generated by cruises. One of the increasingly adopted initiatives for reducing the environmental impact is the reduction of polluting emissions as well as noise pollution in the ports, which is caused by the operation of machinery during the ship scale. As a result, new cold ironing practices are now dependent on the energy supplied by the infrastructure of the port [56, 59, 64].

One of the main environmental impacts of the cruise tourism industry is air pollution, which is influenced by the class of fuel used, the type of engine, the speed of displacement, the maneuvers, and the generation of electricity. The quantity and content can vary significantly, but it is known that most cruises use cheap, sulfur-rich fuels, which are up to 1000 times “dirtier” than the fuel used in road transport (TRT—Transporti e Territorio Srl, 2015). Ship emissions comprise mainly NO_x_, SO_x_, and CO_x_ gases and suspended particles [65].

Estimations from recent years suggest that, on a daily basis, a cruise ship has a carbon footprint greater than 12,000 cars [66]. In addition, the fuel tank that is required by a cruise ship has 2000 times more sulfur than road diesel vehicles, which means that ship emissions during a circuit are the same as those of 350,000 cars [67, 68, 69].

Cruise companies have committed to reducing the current percentage of sulfur (3.5%) to 0.5% by the year 2020; however, there are already companies that work with fuels that contain a rate of 1.5–1.8%. Diesel land vehicles are allowed to emit emissions with a low sulfur content that is estimated to be close to 0.0015%. Governments have been trying to reduce cruise pollution emission since as early as 2005 under the European Community issued Directive 2005/33/EC. This approach proposes that the number of tourism cruise passengers affects the pollution of the environment. Furthermore, other factors such as the capacity of the boats may be related to the quality of the air. Therefore, in the current study, we proposed the follow hypotheses:

H1 *The increase of the number of cruise passengers will influence on the air quality of the port of Barcelona?*

H2 *What is the prediction of the impact of the cruise industry on air pollution of receiving cities?*

## 3 Study area

According to CLIA Europe [1], in 2017, the Spanish port of Barcelona was the most visited cruise of Europe, with an estimated total economic impact of 796 million Euros [36]. In its mission, the port states its commitment to sustainability as a strategic aspect that guarantees the adjustment of the port city and allows for its future sustainability and survival and growth. Spain is one of the most visited destinations for cruise tourism. Table 1 shows the main ports of Spain, and the number of passengers in each. The Port of Barcelona is the first receptor, which will be the case we analyze in this study. It is in the northeast of the Iberian Peninsula next to the Mediterranean Sea, wedged between the new mouth of the Llobregat River and the Barceloneta neighborhood in the city of Barcelona. It is divided into four areas: the commercial port (mainly cargo in containers), citizen port (cruise ships, ferries, leisure areas, Port Vell), energy port, and logistics port. Each of these activities has its own space and is segregated from the others, with its own facilities and specialized personnel. The Port is managed by the Authority of Barcelona, belonging to “Puertos del Estado”.

**Table 1:** Ranking of cruise passengers in Spain.

	Port	Passengers		Port	Passengers
**1**	Barcelona	2,687,365	**15**	Sevilla	21,010
**2**	Baleares	1,958,034	**16**	Ferrol-S, Cibrao	20,061
**3**	Las Palmas	1,105,093	**17**	Huelva	19,573
**4**	Santa Cruz de Tenerife	884,179	**18**	Ceuta	19,259
**5**	Málaga	444,176	**19**	Tarragona	13,445
**6**	Valencia	403,264	**20**	Santander	4801
**7**	Bahía de Cádiz	385,067	**21**	Motril	4570
**8**	Cartagena	187,813	**22**	Avilés	2707
**9**	Vigo	169,093	**23**	Melilla	1224
**10**	A Coruña	126,735	**24**	Castellón	1095
**11**	Alicante	89,000	**25**	Vilagarcía	758
**12**	Bilbao	86,598	**26**	Pasaia	575
**13**	Gijón	32,724	**27**	Bahía de Algeciras	252
**14**	Almería	28,692	**28**	Marín y Ría de Pontevedra	0

Undoubtedly, the normal activity of the Port of Barcelona generates emissions to the atmosphere, contributing to the air pollution of the city. The indicators of emissions of nitrogen oxides and particles in suspension with a diameter of less than 10 μm indicate that port emissions represent between 15 and 20% of the total emissions of the metropolitan region of Barcelona (Table 2).

**Table 2:** Main features of the Port of Barcelona.

**Situation**		**General**	
Latitude	41°21′N	Total area	1,082.15 ha
Length	2°10′E	Docks and berths	22,216 km
**Tides**		Ro-ro ramps	30
Amplitude	125 cm	Openwork	To 16 m
**Entry**		**Storage**	
South Bocana	Orientation: 191.8°	Covered:	203,04 m^2^
	Width: 370 m	Discovered:	5,040,000 m^2^
	Openwork: 16 m	**Dry dock**	
Bocana North	Orientation: 205°	Length:	215 m
	Width: 145 m	Sleeve:	35 m
	Openwork: 11,5 m	Capacity:	50,000 t
Tugs	9 (1.213 kW/2.943 kW)	Cranes	31 (all of containers)

## 4 Method

According to studies by Barcelona City Council, the emissions of nitrogen oxides and particles in suspension with a diameter of less than 10 μm produced within the port area by the accessing vehicles, by civil works for the expansion and maintenance of the infrastructure, by ships and by the machinery used in port operations, account for between 15 and 20% of the total emissions of the metropolitan region of Barcelona. The impact of these emissions on the pollution levels of the city is proportionally lower, contributing below 10%, given that a good part of these emissions are dispersed in the sea. (Port of Barcelona, 2018). The port monitors the air quality by measuring the concentrations of pollutants in the ambient air with a network of high-volume collectors for suspended particles and a network of automatic analyzers for gaseous pollutants. The measurement of these particles began in 1996, when the first high volume collectors were installed to collect samples of this pollutant. Since 2000, PM10 (particulate matter) particles have been measured with an equivalent diameter of less than 10 μm and, subsequently, PM2.5 particles with an equivalent diameter of less than 2.5 μm have also been measured. According to the United States Environmental Protection Agency, PM stands for particulate matter (also called particle pollution), the term for a mixture of solid particles and liquid droplets found in the air. Some particles such as dust, dirt, soot, and smoke, are large or dark enough to be seen with the naked eye. Others are so small they can only be detected using an electron microscope. Particle pollution includes PM10 inhalable particles with diameters that are generally ≤10 micrometers and smaller and PM2.5 fine inhalable particles, with diameters that are generally 2.5 micrometers. To understand how small 2.5 micrometers is, think about a single hair from your head. The average human hair is about 70 micrometers in diameter, making it 30 times larger than the largest fine particle.

The Port has two catchment networks, one manual and the other automatic. The manual network consists of eight collectors of high-volume sequential particles located at five stations. In each of these stations, there is a sensor with a PM10 particle head and three have a second sensor with a PM2.5 particle head. In addition, the port also manages two high volume collectors of total suspended particles (PST) that are used exclusively to measure the concentrations of soy allergens. The PM10 sensor located in Port Vell generates the measurements that are used to officially assess the air quality in the city of Barcelona.

The automatic network consists of three stations equipped with different automatic gas contamination analyzers, such as nitrogen dioxide (NO_2_), sulfur dioxide (SO_2_), ozone (O_3_), and benzene (C_6_H_6_)) Two of these are fixed cabins, ZAL 2 Prat, which only measures NO_2_, and Darsena Sur, which only measures SO_2_, and the third is the Mobile Unit, currently located in Port Vell, which measures all of the above-mentioned pollutants.

This research aimed to analyze the relationship between the arrival of tourists aboard the cruise ships and the quality of the air. For this reason, the aggregated data of passengers of the annual accumulated tourist cruises for the period 2006–2016 and the number of annual accumulated cruises of the same period were also incorporated into the study. We also included a cruise capacity indicator that was obtained by dividing the number of passengers by the number of cruise ships docked in the year. This indicator gave us an idea of the average size of the ships that moor in the Port of Barcelona and allowed us to introduce the size criterion of the vessel to later check if this had any impact on environmental emissions.

We selected the Port of Barcelona as our case study as it is one of the main recipients of cruise tourism in Europe. The data were taken from the environmental quality indicators closest to the Port of Barcelona, by extracting the monthly series of data from the month of January 2006 to the month of December 2017.

As Trozzi and Vaccaro [70] indicate, in the maritime activity it is customary to distinguish between different phases, the first when the ship approaches and docks in the port; the second the hotel business in the port; the third the exit from the port. After its arrival at the port, the ship continues to emit on the dock (in the accommodation phase). This is due to the need to generate energy for lighting, heating, cooling, ventilation, etc. The measurements incorporated in the study were levels of SO_2_ (μg/m³), NO (μg/m³), NO_2_ (μg/m³), O_3_ (μg/m³), and CO (mg/m³). Furthermore, the ICQA (Catalonian Index of Quality of Air) indicator was added to the time series of data, and the information corresponding to the period 2006–2016 was also included. The methodology to select the measures has been developed in the framework of MEET Project (Methodologies for estimating air pollutant emissions from transport), specifically the pollutant taken into consideration are Nitrogen oxides (NOx), Sulfur oxides (SOx), Carbon monoxide (CO), Volatile Organic Compounds (VOC), Particulate matter and Carbon dioxide (CO2). These measures have been also used to measure the environmental impact in the studies of Caríc, and Trozzi and Vaccaro [5, 70].

**Fig. 1:**
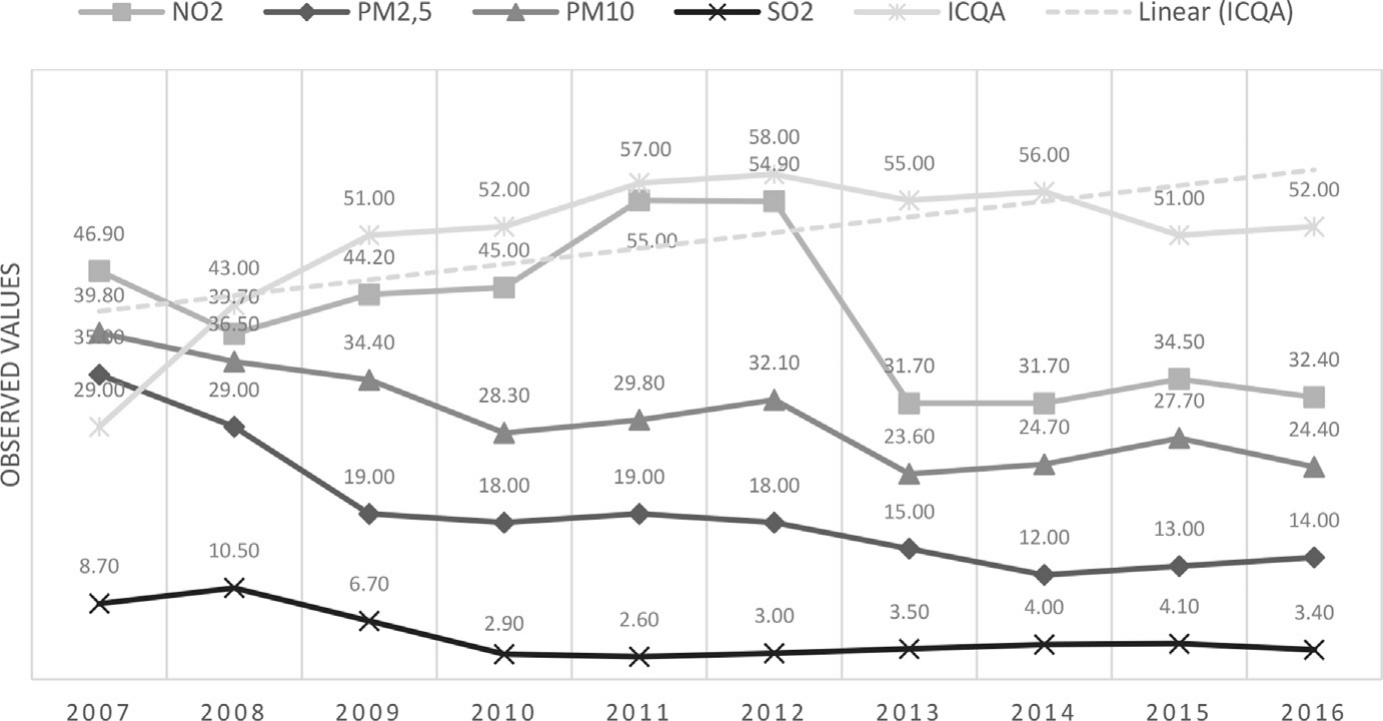
Evolution of the indicators of air pollution in the port of Barcelona (2006–2017).

Source: the authors.

In total, there were 144 monthly data series of each one of these variables. Data that were not available were replaced by the nearest observatory. Fig. 1 shows the evolution of the index along the time and tendency. Most of the indicators decreased over time and the quality of the air index improved. The cruise activity measures were the monthly time series from January 2006 to December 2017 of the number of visiting passengers (144) and the total number of cruise ships docked and the capacity of the cruise ships were used for the study. To facilitate a comparison with the other variables, the arithmetic mean was calculated.

## 5 Results

We conducted multiple tests to evaluate the environmental impact of cruise tourism in the Port of Barcelona. Firstly, it was observed the correlation between the variables of the study (Table 3).

**Table 3:** Correlation between variables.

Correlations							
		MONTH	YEAR	SO2 (μg/m³)	NO (μg/m³)	NO2 (μg/m³)	O3 (μg/m³)
MONTH	Pearson Correlation	1	.000	-.093	.115	-.138	-.172*
	Sig. (2-tailed)		1.000	.267	.170	.099	.039
	N	144	144	144	144	144	144
YEAR	Pearson Correlation	.000	1	-.809**	-.127	-.320**	.103
	Sig. (2-tailed)	1.000		.000	.131	.000	.218
	N	144	144	144	144	144	144
SO2 (μg/m³)	Pearson Correlation	-.093	-.809**	1	.101	.375**	-.053
	Sig. (2-tailed)	.267	.000		.228	.000	.525
	N	144	144	144	144	144	144
NO (μg/m³)	Pearson Correlation	.115	-.127	.101	1	.715**	-.849**
	Sig. (2-tailed)	.170	.131	.228		.000	.000
	N	144	144	144	144	144	144
NO2 (μg/m³)	Pearson Correlation	-.138	-.320**	.375**	.715**	1	-.602**
	Sig. (2-tailed)	.099	.000	.000	.000		.000
	N	144	144	144	144	144	144
O3 (μg/m³)	Pearson Correlation	-.172*	.103	-.053	-.849**	-.602**	1
	Sig. (2-tailed)	.039	.218	.525	.000	.000	
	N	144	144	144	144	144	144
CO (mg/m³)	Pearson Correlation	-.013	-.800**	.850**	.208*	.413**	-.109
	Sig. (2-tailed)	.880	.000	.000	.012	.000	.194
	N	144	144	144	144	144	144
ICQA	Pearson Correlation	.296**	.349**	-.437**	.067	-.298**	-.316**
	Sig. (2-tailed)	.000	.000	.000	.422	.000	.000
	N	144	144	144	144	144	144
PASSENGERS	Pearson Correlation	.400**	.044	-.056	-.644**	-.574**	.606**
	Sig. (2-tailed)	.000	.603	.503	.000	.000	.000
	N	144	144	144	144	144	144
CRUISES	Pearson Correlation	.428**	-.032	-.010	-.584**	-.468**	.550**
	Sig. (2-tailed)	.000	.700	.903	.000	.000	,000
	N	144	144	144	144	144	144

*Correlation is significant at the 0.05 level (2-tailed). **Correlation is significant at the 0.01 level (2-tailed).

The results indicate that there is a strong correlation between the indicator variables of environmental impact, so we proceed to group them into a factor named environmental indicators (EI) through a factorial exploratory analysis. This index includes the effects of NO (μg/m³), NO2 (μg/m³), and O_3_ (μg/m³). Table 4 shows the communalities of the three variables grouped, with a variance of 81.631% explained.

**Table 4:** Communalities of the factor Environment Index.

	Initial	Extraction
NO (μg/m³)	1	0.904
NO_2_ (μg/m³)	1	0.718
O_3_ (μg/m³)	1	0.827

With the information available, the relationships between the dependent (EI) and the independent variables, (passenger number and cruise capacity) were analyzed. Initially, a Pearson correlation analysis was conducted to identify the cases where passenger traffic was related to the air quality of the port. After that, we performed a correlation analysis including the factor index, as shown in Table 5.

**Table 5:** Correlation analyses.

	Month	Year	ICQA	Passengers	Cruises	EI
Month	1					
Year	0	1				
ICQA	0.296**	0.349**	1			
Passengers	0.400**	0.044	−0.004	1		
Cruises	0.428**	−0.032	−0.035	0.891**	1	
EI	0.061	−0.198 *	0.04	−0.674**	–0.593**	1

**Correlation is significant at the 0.01 level (2-tailed). *Correlation is significant at the 0.05 level (2-tailed). EI: Environment Index; ICQA: Catalonian Index of Quality of Air.

**Fig. 2:**
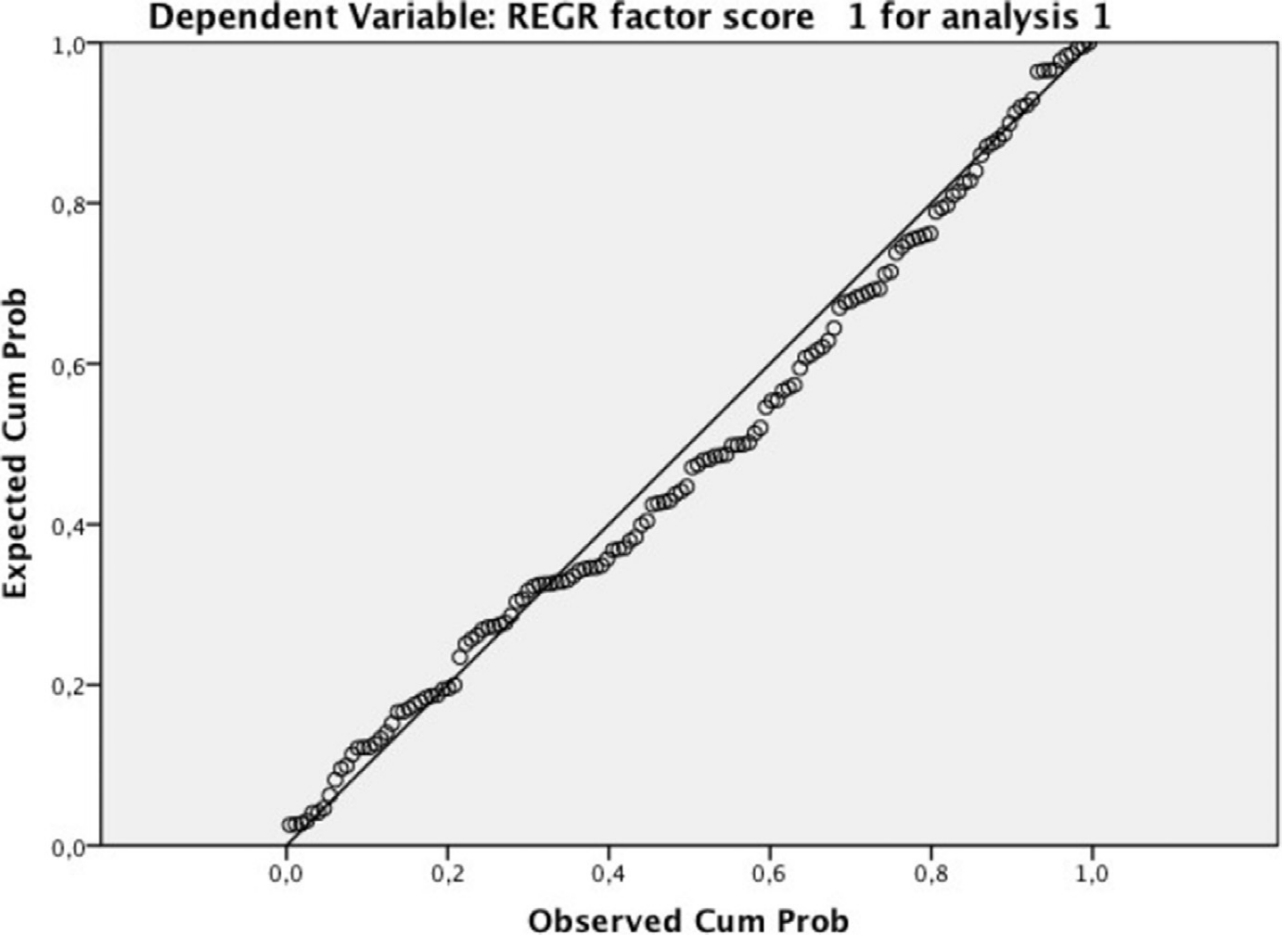
Normal P-P plot of regression standardized residual.

**Fig. 3:**
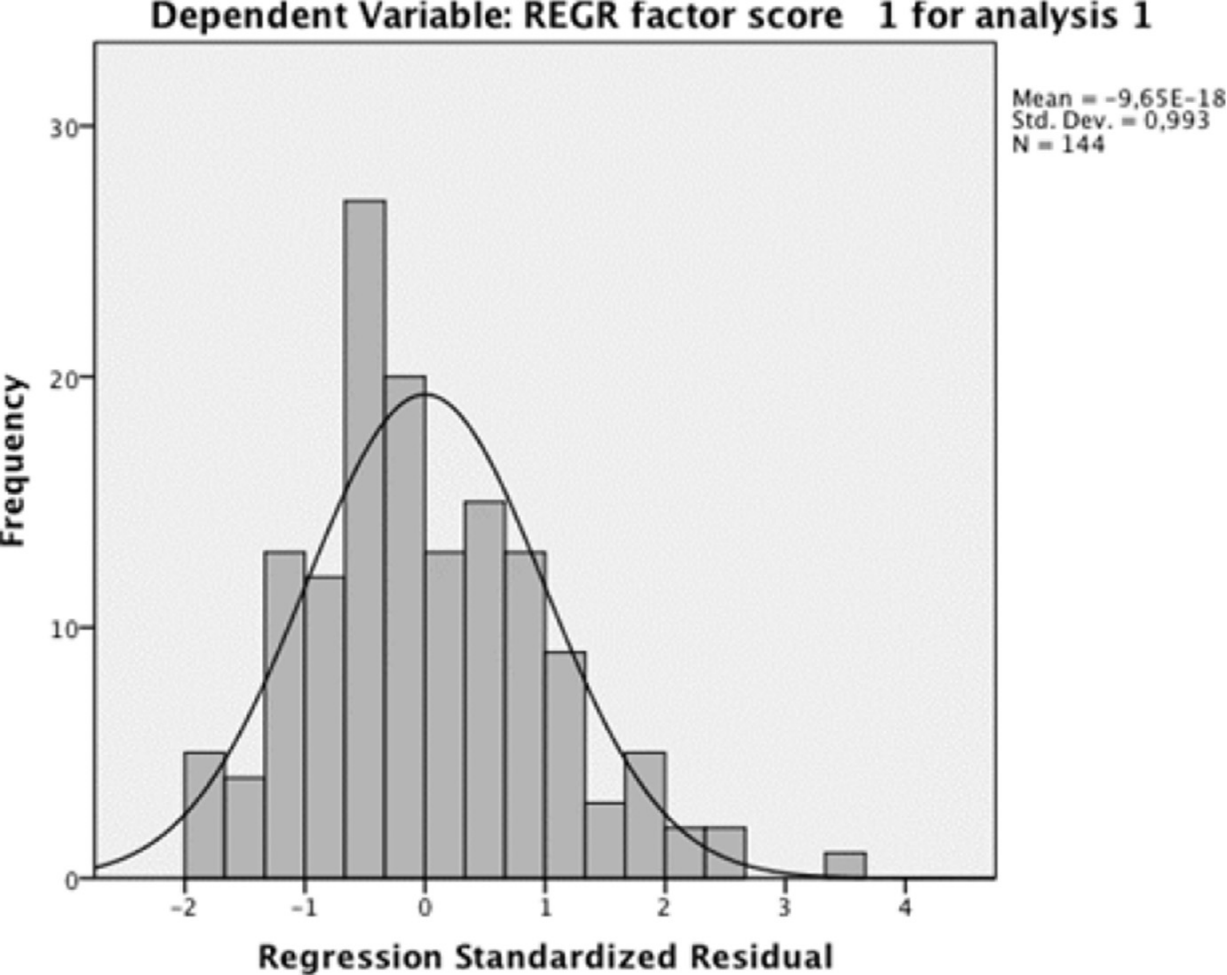
Histogram.

Next, the main assumptions required to perform a linear regression analysis were analyzed. First, a scatterplot of the Environment Index concentration against the average passenger number was plotted. A visual inspection of the scatterplot indicated a linear relationship between the variables (Fig. 2). After that, in relation to the outlier analysis, the case wise diagnostics showed three cases where the anomaly index was higher than three. These three cases were deleted, as they did not represent the target population. There was homoscedasticity, as assessed by a visual inspection of a plot of standardized residuals versus the standardized predicted values. Additionally, the residuals were normally distributed as assessed by the visual inspection of a normal probability plot (Fig. 3). In relation to the independence of the residuals, a Durbin–Watson analysis, assessed this.

**Fig. 4:**
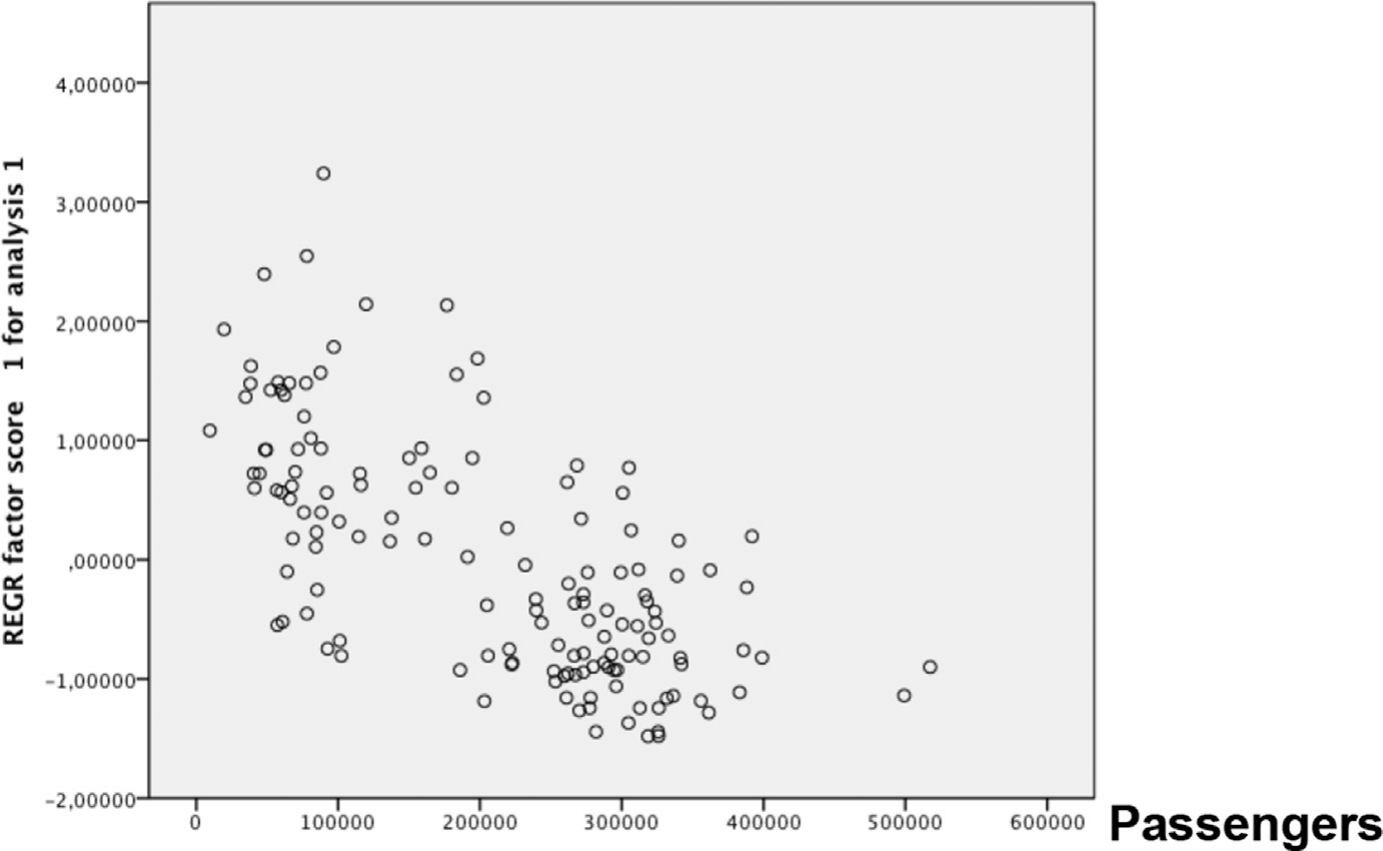
Regression analysis.

Once the assumptions were confirmed, a linear regression was run to determine the effect of the average passenger number on the Environment Index concentration. Table 6 shows the variables used for the analysis, Table 7 presents the model summary, and Table 8 contains the coefficients of the linear relation. The Fig. 4 shows graphically the regression between variables, passengers and factor score of environmental impact.

**Table 6:** Variables entered/removed.

Model	Variables Entered	Variables Removed Method
1	Cruises, Passengers	Enter

All requested variables entered. Dependent variable: REGR factor score 1 for analysis 1.

**Table 7:** Model summary.^b^

Model	R	R Square	Adjusted R Square	Std. Error of the Estimate	Durbin-Watson
1	0.674^a^	0.454	0.446	0.74421423	0.984

a Predictors: (Constant), cruises, passengers.

b Dependent variable: REGR factor score 1 for analysis 1.

**Table 8:** Coefficients of the model.

Coefficients^a^								
Model		Unstandardized Coefficients		Standardized Coefficients	t	Sig.	95.0% Confidence Interval for B	
		B	Std. Error	Beta			Lower Bound	Upper Bound
1	(Constant)	1.211	0.134		9.013	0	0.945	1.477
	Passengers	−6.15 × 10^−6^	0	−0.705	−5.145	0	0	0
	Cruises	0.001	0.004	0.036	0.261	0.794	−0.007	0.009

a Dependent variable: REGR factor score 1 for analysis 1.

According to Table 7, the average passenger numbers accounted for 44.6% of the variation of the Environment Index concentration with an adjusted R^2^ = 44.6%, a medium size effect according to Cohen [71].

Finally, an ANOVA analysis was performed to evaluate the statistical significance of the model. According to Table 9, the average passenger number statistically significantly predicted the Environment Index concentration, F (2, 141) = 58,595, *p* < 0.0005.

**Table 9:** ANOVA test results.

	ANOVA^b^					
	Model	Sum of Squares	df	Mean Square	F	Sig.
1	Regression	64.906	2	32.453	58.595	0.000^a^
	Residual	78.094	141	0.554		
	Total	143	143			

a Predictors: (Constant), cruises, passengers.

b dependent variable: REGR factor score 1 for analysis 1.

The prediction equation was:

$$\text{EI}\;=-6.15\text{×}10^{-6}+0.036\text{×Passengers.}$$

The prediction of the Environment Index was statistically significant, F (2, 141) = 58.595, *p* < 0.0005, accounting for 44.6% of the variation in the Environment Index concentration with an adjusted R^2^ = 44.0%. This implies a high size effect according to Cohen [66]. Therefore, the results allowed us to confirm the first hypothesis. On the other hand, the coefficient of cruises (Table 8) was not statistically significant, so we discarded the second hypothesis that linked the cruise capacity and the Environment Index.

## 6 Discussion & conclusions

The cruise tourism industry is implementing new consumer trends, so companies in the sector have to adapt their distribution systems to allow significant increase. On the other hand, society is demanding sustainable long-term businesses that are respectful of the environment and minimize its negative externalizations. Additionally, the development of regulations has increasingly limited the discretion of companies by imposing criteria regarding environmental sustainability. International regulations are changing to better and further protect the environment and the cycle of waste, water, and emissions.

**Fig. 5:**
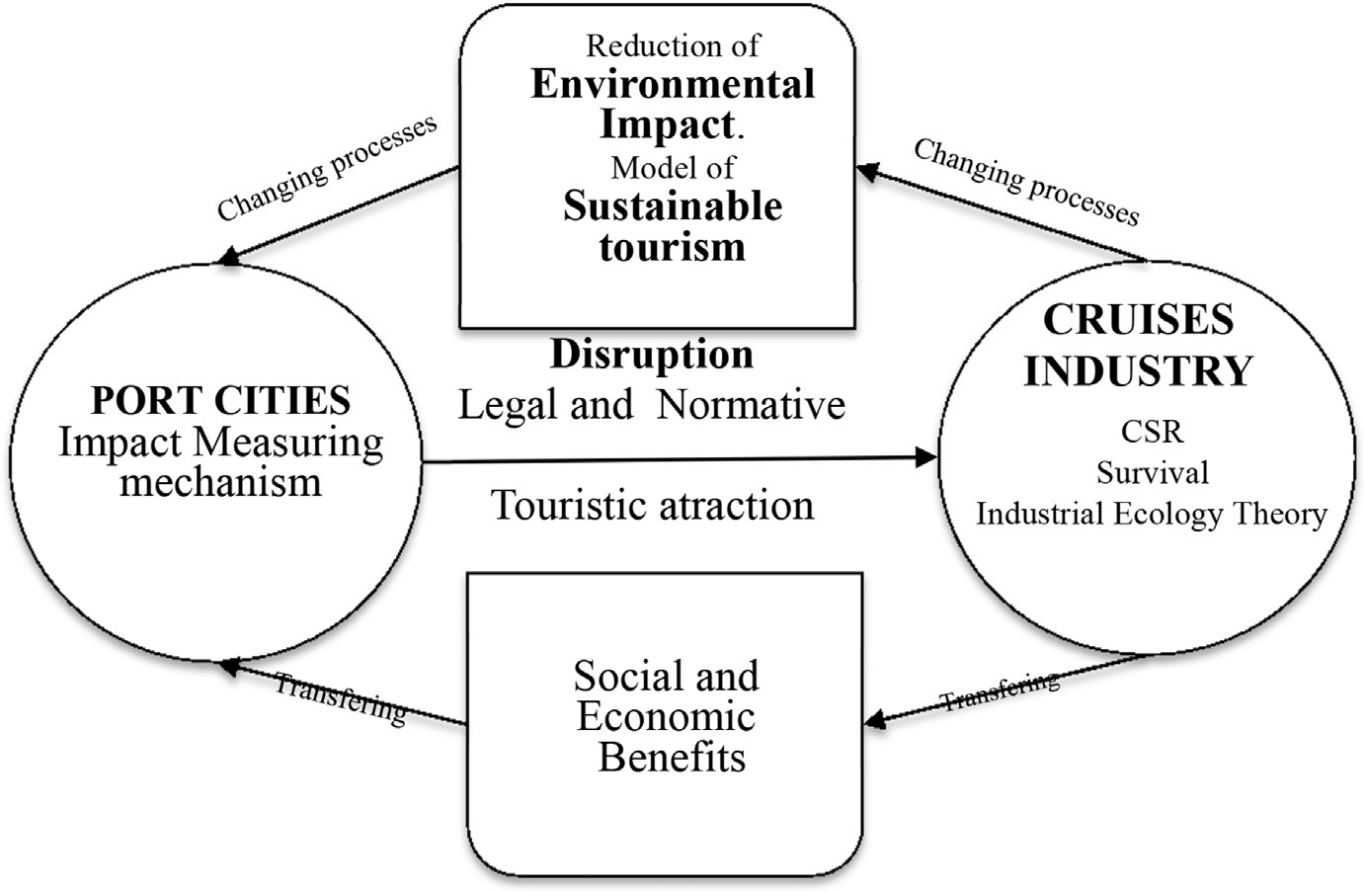
Cruise Industry Sustainability model.

Source: the authors.

The cruise industry is increasing its commercial interest, but ships must be adapted to the new laws. Obviously, the limitations that have been established for other transport companies such as diesel vehicles will be extended to ships. Therefore, according to the model proposed in Fig. 5, the cruise industry must have a positive balance between economic benefits and its environmental impact on port cities. This Figure shows a sustainability model for the cruise industry that guarantees this positive balance. Additionally, in this paper, we showed that since passenger volumes have grown enormously in the last 10 years in Barcelona, the port needs to create controls to understand the impact of emissions. This growth also has impacts on the environmental indicators of the city, where a significant relationship between the number of passengers and the environmental conditions of the environment of the post was observed.

In addition, the sustainability of this industry includes exact knowledge on the part of the tourist, in accordance with the GHG Protocol or ISO 14064-1, of the carbon footprint generated by cruise ships. We need consider that tourism has created situations that have pushed society to explore other alternatives by closing channels that generate problems in the environment such as noise or emissions derived from the massive influx of tourists.

Through this investigation, we detected that the cruise industry requires new academic advances in different aspects. The trends that this industry will develop under in the coming years, including the expected increase in demand, will force shipping companies to modify their organizational structures, making changes to adapt to the new market situation. On the other hand, governments of the countries and receiving cities of the cruise ships will have to create new norms that demand the sustainability of this tourist activity, forcing companies to modify their business vision.

We formulated two research question, firstly we want to know if there is a significant relationship between number of passenger/cruises and air pollution. The results of our research contribute to the understanding of the environmental impact on the air of the cruise tourism industry. Thanks to the regression analysis, we observe a linear relationship between number of passenger and air pollution. Additionally, it is possible to predict this effect from forecasts of the number of tourists. In other words, information on the environmental impact can be anticipated once the number of passengers that will visit the cities is known. This forecast could help mitigate the negative effects, thus avoiding very high levels of pollution in cities. Our second research question aims to predict the impact of the cruises in the air quality. Although other works have addressed research on air pollution and ships in tourism [72, 73, 74], or in other industries [75] there is no research to evaluate this effect by time series over such a long period of time (11 years) and also provide a prediction based on in the regression analysis carried out.

As second contribution we formulate the predictive equation:

$$\text{EI}=-6.15\times 10^{-6}+0.036\times \text{no}\;\text{of}\;\text{passengers.}$$

Where EI is the monthly environmental index including the effects of CO2 emissions, PM10 and PM2.5 particles, NO2 and SO2. So, thanks to this predictive equation, the port of the city of Barcelona knows the impact of the traffic of futures cruises and take measures to reduce this pollution.

As a third contribution of this work, the results allowed us to identify a sustainable model of relationships between the cruise industry and the host cities, as shown in Fig. 5. This presents an opportunity to develop new ideas of sustainability that can be used as a reference for decision-making in shipping companies, based on the Industrial Ecology Theory.

The research methodology allows us to acknowledge a fourth contribution. We verified that ports have systems to measure the polluting emissions of port activities. However, these mechanisms are insufficient. The development of new measurement systems could help to determine the emissions of the ships in each port exactly and for each ship, thus allowing the discrimination of the impact of each cruise individually.

The analyzed information shows the correlations between cruise ship emissions and their effects on climate change, demonstrating that this industry needs to invest in innovation and technology to improve its competitiveness and sustainability. The new machinery will allow it to be more sustainable; meanwhile, it will be necessary to manage situations regarding old ships with obsolete machinery, high levels of CO_2_ emissions, and a strong environmental impact.

In addition, we consider it necessary for cruise ships to report their CO_2_ footprint to each passenger, as is usual practice in other means of transport or in other tourist activities. This will allow the necessary awareness to invest further in social responsibility, be more ecological and sustainable, and rely on the use of clean energy. Therefore, we conclude that cruise companies and port city authorities must make plans to reduce and offset current CO_2_ emissions. The city of Barcelona is an example of how the proposed model can be used to improve the quality of life of port city inhabitants and makes cruise tourism sustainable.

Finally, regarding to limitations and futures research lines, this paper presents some limitations derived from the methodology used. First, the measurement of the impact of tourism on air pollution may be incomplete and it would be convenient to include other relevant aspects of this impact, such as the issue of waste generated by the ships' visit to the ports, secondly the effect on the waters of the port, the pollution observed in this environment. Third, visitors' carbon footprint could be included, calculated by adding up the total environmental impact. Another limitation is that the work, by focusing on the specific case of the Spanish Port of Barcelona, may be incomplete and these same effects should be tested in other important ports of cruise tourism.

## Declarations

### Author contribution statement

Ignacio Ruiz-Guerra: Conceived and designed the experiments; Contributed reagents, materials, analysis tools or data.

Valentín Molina-Moreno: Performed the experiments; Analyzed and interpreted the data; Contributed reagents, materials, analysis tools or data; Wrote the paper.

Francisco J. Cortés-García: Analyzed and interpreted the data; Wrote the paper.

Núñez-Cacho Pedro: Conceived and designed the experiments; Analyzed and interpreted the data.

### Funding statement

This work was supported by Research Project ECO2017-84138-P MINECO, AGENCIA ESTATAL DE INVESTIGACIÓ“N and FEDER (EU).

### Competing interest statement

The authors declare no conflict of interest.

### Additional information

No additional information is available for this paper.

## Appendix ASupplementary data

DATABARNIA2018 DATABARNIA2018
